# CRISPR genome editing using a combined positive and negative selection system

**DOI:** 10.1371/journal.pone.0321881

**Published:** 2025-05-06

**Authors:** Ishrya Sharma, Kerisa Hall, Shannon Moonah

**Affiliations:** 1 Department of Medicine, University of Florida, GainesvilleFlorida, United States of America; 2 Department of Molecular Genetics and Microbiology, University of Florida, GainesvilleFlorida, United States of America; Central University of Andhra Pradesh, INDIA

## Abstract

The clustered regularly interspaced short palindromic repeats (CRISPR)/Cas system is a powerful genome editing tool that has revolutionized research. Single nucleotide polymorphisms (SNPs) are the most common form of genetic variation in humans. Only a subset of these SNPs has been shown to be linked to genetic diseases, while the biological relevance of the majority remains unclear. Investigating these variants of unknown significance could provide valuable insights into their roles in biological processes, disease susceptibility, and treatment responses. While CRISPR/Cas has emerged as a transformative technology, its ability to make single nucleotide substitutions remains a significant limitation. Other techniques in single nucleotide editing, such as base editing and prime editing, offer promising possibilities to complement CRISPR/Cas systems, though they also have their own limitations. Hence, alternative approaches are necessary to overcome the limitations of CRISPR. Here, to improve the feasibility of generating single base edits in the genome, we provide a protocol that introduces a multiple expression and dual selection (MEDS) system, which, alongside CRISPR, utilizes the opposing roles of cytosine deaminase/uracil phosphoribosyltransferase (CD/UPRT) for negative selection and neomycin phosphotransferase II (NPT II) for positive selection. As a proof of concept and to demonstrate feasibility of the method, we used MEDS, along with traditional CRISPR-Cas9, to generate sickle hemoglobin by introducing a point mutation (A → T) in the sixth codon of the hemoglobin beta gene.

## Introduction

The clustered regularly interspaced short palindromic repeat (CRISPR) system has become a revolutionary and widely utilized tool for genome editing across various organisms and cell lines. The CRISPR/Cas system is used in a significant number of applications, including pharmacogenomics, personalized medicine, genome-wide association studies, and gene therapy for genetic disorders. The CRISPR/Cas system is based on the interaction between an engineered single guide RNA (sgRNA) that is homologous to a target DNA sequence. These easily engineered sgRNA then bind to Cas9, steering the nuclease to the target DNA site, where a double-stranded break (DSB) is made [1,2]. Following the double-stranded break, natural repair processes take over, leading to the insertion of specific DNA sequences by the initiation of homology directed repair (HDR) or insertions or deletions (indels) by the nonhomologous end joining (NHEJ) pathway [3]. Traditional CRISPR/Cas techniques, while powerful, lack the efficiency and feasibility needed to alter single nucleotides.

Single nucleotide polymorphism (SNP) is a type of genetic variation involving a single nucleotide change in the DNA sequence. This change can lead to a missense mutation in which the genetic alteration encodes a different amino acid. Single amino acid changes in proteins may lead to disruption in protein folding, stability or function. Hence, protein variants can have significant impacts on human health [4]. For example, sickle cell disease and cystic fibrosis are prevalent disorders that result from protein variants with disrupted activity and are associated with significant health challenges and potential complications [5,6]. In addition, SNPs in the APOE gene are associated with increased risk in Alzheimer’s and Cardiovascular Diseases [7,8]. That said, most protein variants remain uncharacterized, posing a major challenge in understanding their impact on human health. Of the millions of known missense variants, only about 2% have been definitively classified as benign or pathogenic, leaving the vast majority with unknown clinical significance [9]. Developing efficient and effective tools to investigate how variants influence protein function will be essential to expanding our understanding of their molecular and clinical consequences.

Two CRISPR/Cas-derived methods have been developed to generate single-nucleotide substitutions in the genome, namely base editors and prime editors. Base editors consist of a catalytically impaired Cas nickase fused to a nucleobase deaminase enzyme [10]. Currently, there are three categories of base editors: cytosine base editors, which modify C • G-to-T • A base pairs; adenine base editors, which convert A • T-to-G • C base pairs; and the newly created C-to-G base editors, which induce C • G-to-G • C transversions [2,11]. Despite their potential, there are several limitations to base editors that impact their widespread use. First, a major limitation of base editing is that this method can only create half of all possible point mutations. In contrast to other CRISPR-Cas9 genome editing tools, base editing does not generate insertions or deletions [12]. Second, base editing can have unique, non-specific effects that are not typically seen with traditional CRISPR/Cas9 methods using HDR. For example, bystander mutations can result from base editors deaminating nucleotides within a 4–5 nucleotide window, where nearby C or A nucleotides adjacent to the target may also be converted [2]. When studying the effects of single-nucleotide changes in the genome, it is essential to ensure that no other nucleotides in the surrounding genomic area are affected to accurately replicate a naturally occurring mutated genomic environment. There are also reported significant off-target activity and limited efficiency with genome editing using base editors [13–15]. Prime editors comprise of a Cas9 nickase fused to a reverse transcriptase (RT) and a prime editing guide RNA (pegRNA) [16]. Prime editors can generate virtually any point mutation, but also face their own limitations, including relatively high costs and significantly low, unpredictable, and variable editing efficiency compared to other genome editing tools. For example, some cell lines show efficiency rates below 20% [2], and this rate declines further in primary cells. Therefore, alternative strategies for single base editing are needed.

We developed a multiple gene expression and dual selection (MEDS) system, designed for use with traditional CRISPR/Cas9 technology to allow feasible single nucleotide substitutions in the genome. Here, we describe a protocol that combines MEDS with CRISPR/Cas9 and as proof of feasibility, we mutated adenine to thymine (A to T) in the sixth codon of the beta-globin gene.

## Materials and methods

The protocol described in this peer-reviewed article is published on protocols.io (https://dx.doi.org/10.17504/protocols.io.j8nlk99r6v5r/v1) and is included as supporting information (S1 File) file with this article.

## Expected results and discussion

**Fig 1:**
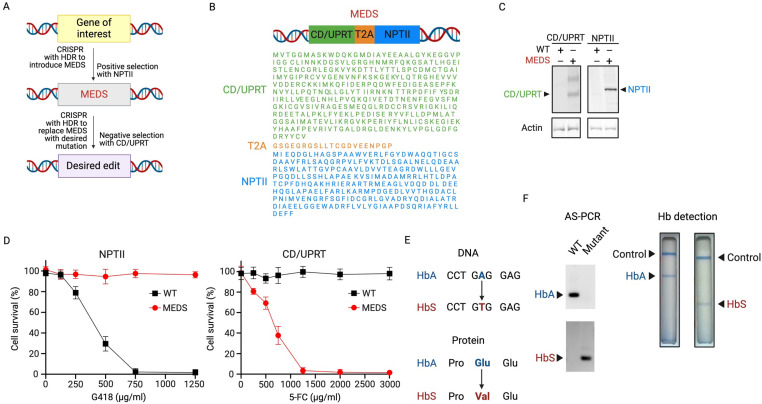
CRISPR/Cas9 genome editing using MEDS (A) Schematic representation of CRISPR genome editing strategy. (B) Amino acid sequence of the MEDS system. (C) Immunoblot analysis showing CD-UPRT and NPT II protein expression. Actin was used as a loading control. (D) Cell survival of WT and MEDS erythrocyte precursor cells 7 days after drug selection. (E-F) Detection of wild type and sickle hemoglobin using Allele-Specific PCR and Sickle Scan assays. Data are representative of 3 independent experiments. Data represent means and SD. Abbreviations: MEDS, multiple gene expression and dual selection; HDR, homology directed repair; CD/UPRT, cytosine deaminase/uracil phosphoribosyltransferase; NPT II, neomycin phosphotransferase II; NPT II; T2A, 2A self-cleaving peptide; WT, wild type, G418, geneticin; 5-FC, 5-fluorocytosine (5-FC); AS-PCR, allele-specific polymerase chain reaction; Hb, hemoglobin.

MEDS consists of cytosine deaminase/uracil phosphoribosyltransferase (CD/UPRT) for negative selection and neomycin phosphotransferase II (NPTII) for positive selection (Fig 1A, B). CD/UPRT converts the non-toxic 5-fluorocytosine (5-FC) into toxic metabolites 5-fluorouracil (5-FU) and 5-fluorouridine monophosphate (5-FUMP) [17]. CD/UPRT was found to be more potent than other negative selection enzymes. Cytosine deaminase alone and thymidine kinase can alternatively be used to render cells susceptible to negative selection by 5-FC and ganciclovir (GCV), respectively. However, these methods are less efficient than using the CD/UPRT combination, as observed in various human cell types, including human colorectal cells, pancreatic cells, and breast cancer cell lines. Additionally, thymidine kinase activity is often restricted to actively dividing cells, which further limits its use. CD/UPRT selection is not dependent on the cell cycle, which allows for consistent and efficient negative selection across a broader range of cell types, including those that are not actively dividing. NPT II allows resistance to certain aminoglycoside antibiotics, such as neomycin, kanamycin, and G418 (geneticin) [18]. Although G418 was used here for positive selection, other selection markers, such as blasticidin, hygromycin B, puromycin, or zeocin, can be used by replacing NPTII with the appropriate resistance gene.

Our approach of creating SNPs in the genome using CRISPR/Cas9 with HDR follows two main steps (Fig 1A). Following both rounds of transfection, the cells were passaged for 3 weeks (passage 8) prior to G418 or 5-FC selection to dilute out cells containing the donor episomal plasmid. First, the beta subunit of normal adult hemoglobin A (HbA) gene was knocked out and replaced with MEDS by co-transfecting TF-1 erythroblast cells with Cas9/gRNA and MEDS donor plasmids. CRISPR/Cas9-edited cells were selected for using G418 positive selection. The presence of functional CD/UPRT and NPT II was confirmed through immunoblot analysis and subsequent cell viability assays (Fig 1C, D). Following verification of MEDS insertion, MEDS was replaced with the hemoglobin S (HbS) gene using a second co-transfection of Cas9/gRNA and donor plasmid containing the HbS gene. Cells containing MEDS were selected against using 5-FC negative selection. Allele-specific polymerase chain reaction (AS-PCR) is a commonly used technique for detecting SNPs [19–22]. AS-PCR was performed to select for edited clones, which were then confirmed by Sanger sequencing (Fig 1E, F). Sickle Scan is an immunoassay that detects the presence of HbA and HbS proteins [23–25]. The presence of HbS was confirmed using the Sickle Scan assay (Fig 1E, F).

When customizing the MEDS system, the orientation of selection markers is essential for optimal protein expression and function. The recommended order of the MEDS system is to first include the negative selection marker CD/UPRT, followed by the T2A with linker, and lastly, the positive selection marker, such as NPT II. Also, drug selection concentration may vary, so performing a drug kill curve is necessary to determine the appropriate concentration for effective cell elimination.

CRISPR/Cas genome editing results in a mixed pool of edited cells, including homozygous mutants, heterozygous mutants, or unedited homozygotes [26]. To generate cell lines with a specific edit, clonal expansion of single cells is needed. Commonly used methods to generate single clones include limiting dilution, isolation using cloning cylinders, and fluorescence-activated cell sorting (FACS). Our protocol includes both limiting dilution and cloning cylinder techniques. Limiting dilution involves creating a highly diluted cell suspension to obtain one cell per well [27]. Limiting dilution is often used because of its simplicity, low cost, and high cell viability. Also, limiting dilution may be used across broad cell types, including both adherent and suspension cell lines. However, when there is no selection pressure, limiting dilution may be inefficient and require an excess of resources during clone verification. Using a selection system, such as MEDS can help address the challenges of low efficiency associated with limiting dilution as it selects for the growth of cells that have the desired genetic modifications. Cloning cylinders allow for the isolation of single colonies from a cell culture dish [28]. This method allows for more efficient expansion of clones, as colonies tend to grow faster compared to isolated single cells in limiting dilution. The main limitation is that cloning cylinders can only isolate single colonies from adherent cell lines. As a specialized technique of flow cytometry, FACS enables the sorting of heterogenous cell mixtures, one cell at a time, based on its unique fluorescence properties. FACS is expensive, time consuming, and requires technical expertise. Additionally, cells isolated through FACS may yield cells with low viability because the high pressures used in this method induce stress on the cells. Fluorescent markers, such as green fluorescent proteins (GFP) may be used to sort edited single cells. However, each cell may express different levels of GFP, resulting in varying fluorescent intensities, which makes it difficult to standardize the sorting of positive cells. Of the 12 clones we screened, all 12 (100%) possessed the MEDS sequence in the genome of TF-1 erythroblast cells. That said, efficiency will vary depending on the cell type.

Compared to traditional CRISPR/Cas editing methods, prime and base editing face financial, technical, and practical challenges. Both prime and base editing often require specialized components and equipment that increase experimental costs. In contrast, traditional CRISPR/Cas system requires plasmids that are sometimes available at low cost through plasmid sharing resources such as Addgene [29,30]. Base editing generally yields lower efficiencies compared to the CRISPR-Cas system, ranging widely depending on the specific editor and target from as low as 0.8% to 60% [31,32]. Similarly, prime editing can also be limited by its low editing efficiency [33]. For example, the editing efficiency of Prime Editor 2 (PE2) was below 20% in immortalized cell lines and declined even further in primary cells [34]. In addition, identifying correctly edited single-cell clones can be also time-consuming and expensive with prime and base editing techniques. This is largely due to the extensive screening and validation processes, which might not be practical for some researchers. Utilizing a dual positive and negative selection system such as MEDS can help alleviate the costs, as the cells that are selected for and against contain the desired genomic edits. The most time-consuming aspect of the MEDS system is the duration required for drug selection. However, once the drug selection process is complete, the validation process becomes significantly more time efficient. Both prime and base editing are associated with off-target effects. However, prime editing is considered to have fewer off-target effects compared to CRISPR/Cas systems. The off-target risks associated with CRISPR/Cas systems can be significantly minimized through careful gRNA design [35,36].

DSBs created by Cas proteins lead to either HDR or non-homologous end joining (NHEJ) repair pathways. After a DSB, NHEJ pathway ligates the broken ends and generates insertions or deletions. HDR mainly occurs during the G2 and S phases of the cell cycle, while NHEJ can occur in all phases of the cell cycle. Since HDR is active during these cell cycle phases, this limits its use to actively dividing cell lines. Our method is applicable to a range of cell lines and types, including primary and immortalized cell lines. CRISPR/Cas genome editing using HDR has been successfully reported in several primary cell lines, including primary human mesenchymal stem cells [37], neonatal fibroblast cells [38], hematopoietic stem and progenitor cells [39] (HSPCs) to name a few. Gene editing with the CRISPR/Cas system is a highly effective method for creating mutations in the genome of immortalized cell lines. A limitation of using primary cell lines for CRISPR/Cas editing is that they have a finite lifespan and limited replication capacity. Due to the shortened lifespan of primary cell lines, there can be lower HDR efficiency rates. In this case, researchers can use small molecules that enhance HDR by suppressing the NHEJ pathway, cell cycle synchronization, or stimulating the HDR pathway directly. Several molecules enhance HDR efficiency, including RS-1 [40,41] (RAD51 activator), L755507 [42,43] (a β3-adrenergic receptor agonist), Scr7 [44] (DNA ligase IV inhibitor), and DNA-dependent protein kinase (DNA-PK) inhibitors (NU7026 and NU7441) [45,46].

A number of SNPs are located in non-coding regions, such as promoters, and correlate with disease. SNPs in non-coding regions can alter gene expression by affecting processes such as transcription factor binding, often leading to changes in protein levels, and are therefore worth characterizing [4,47]. MEDS can be used along with CRISPR to insert SNPs in non-coding regions. This approach requires incorporating a constitutive promoter in front of the MEDS sequence to allow expression of the selection markers. For example, cytomegalovirus (CMV), elongation factor 1α promoter (EF1A), and human phosphoglycerate kinase (hPGK) promoters are strong, constitutive elements that initiate transcription in a wide range of cell types, ensuring high-level expression of the desired protein [48,49].

The CRISPR/Cas genome editing system has emerged as a powerful technology. Our protocol presents an opportunity to improve upon existing CRISPR/Cas techniques, potentially addressing some of the challenges associated with making single-base edits in the genome.

## Supporting information

S1 File Step-by-step protocol, also available on protocols.io. (PDF)
