# Supplementary material for: CRISPR genome editing using a combined positive and negative selection system
Source: PLoS One. 2025 May 6;20(5):e0321881. doi: 10.1371/journal.pone.0321881 (PMC12054870; doi:10.1371/journal.pone.0321881)
Supplement: S1 File — (PDF) [file pone.0321881.s001.pdf]

CRISPR genome editing using a combined positive and negative selection system

RESERVED DOI:
10.17504/protocols.io.j8nlk99r6v5r/v1

Ishrya Sharma[1], Kerisa Hall[1], Shannon Moonah[1,2]

[1]Department of Medicine, University of Florida, Gainesville, FL, USA;
[2]Department of Molecular Genetics and Microbiology, University of Florida, Gainesville, FL, USA

Moonah Lab
UF

Protocol Info: Ishrya Sharma, Kerisa Hall, Shannon Moonah . CRISPR genome editing using a combined positive and negative selection system. **protocols.io** **https://protocols.io/view/crispr-genome-editing-using-a-combined-positive-an-du4v6yw6**

Created: December 15, 2024

Last Modified: February 19, 2025

Protocol Integer ID: 115573

Keywords: genome editing, CRISPR, Cas, homology-directed repair, single nucleotide polymorphism

Funders Acknowledgements:
National Institutes of Health
Grant ID: R01DK131313

Abstract

The clustered regularly interspaced short palindromic repeats (CRISPR)/Cas system is a powerful genome editing tool that has revolutionized research. Single nucleotide polymorphisms (SNPs) are the most common form of genetic variation in humans. Only a subset of these SNPs has been shown to be linked to genetic diseases, while the biological relevance of the majority remains unclear. Investigating these variants of unknown significance could provide valuable insights into their roles in biological processes, disease susceptibility, and treatment responses. While CRISPR/Cas has emerged as a transformative technology, its ability to make single nucleotide substitutions remains a significant limitation. Other techniques in single nucleotide editing, such as base editing and prime editing, offer promising possibilities to complement CRISPR/Cas systems, though they also have their own limitations. Hence, alternative approaches are necessary to overcome the limitations of CRISPR. Here, to improve the feasibility of generating single base edits in the genome, we provide a protocol that introduces a multiple expression and dual selection (MEDS) system, which, alongside CRISPR, utilizes the opposing roles of cytosine deaminase/uracil phosphoribosyltransferase (CD/UPRT) for negative selection and neomycin phosphotransferase II (NPT II) for positive selection. As a proof of concept and to demonstrate feasibility of the method, we used MEDS, along with traditional CRISPR-Cas9, to generate sickle hemoglobin by introducing a point mutation (A → T) in the sixth codon of the hemoglobin beta gene.

Materials

Reagents

1. Complete RPMI media: 500 mL RPMI, HEPES (Gibco, USA, Catalog # 22400089), 50 mL (10%) Heat Inactivated Fetal Bovine Serum (Gibco, USA, Catalog # 10082147), 100 mL (2 ng/mL) Granulocyte-Macrophage Colony-Stimulating Factor human (Sigma-Aldrich, USA, Catalog # G5035-5UG), and 5 mL (1X) Pen/Strep (Gibco, USA, Catalog # 15140122).
2. Fisherbrand™ Surface Treated Sterile Tissue Culture Flasks, Vented Cap (Fisher Healthcare, USA, Catalog # FB012937).
3. Scienceware® cloning cylinders, polystyrene (Sigma-Aldrich, USA, Catalog # Z370789).
4. Trypsin-EDTA (0.05%), phenol red (Gibco™, USA, Catalog # 25300054).
5. Fisherbrand™ Surface Treated Tissue Culture Dishes (Fisher Scientific, USA, Catalog # FB012924).
6. Cell counting slides (Bio-Rad, USA, Catalog # 1450015).
7. Opti-MEM™ I Reduced Serum Medium (Gibco, USA, Catalog # 31985070).
8. pcDNA3 Mammalian Expression Vector (Invitrogen, USA, Catalog # V79020).
9. Donor plasmid + Cas9/guide RNA plasmids
10. FuGENE® HD Transfection Reagent (Promega, USA, Catalog # E2311).
11. Corning™ Costar™ 12-well Clear TC-treated Multiple Well Plates, Individually Wrapped, Sterile (Fisher Healthcare, USA, Catalog # 07-200-82).
12. Geneticin™ Selective Antibiotic (G418 Sulfate) (50 mg/mL) (Gibco, USA, Catalog # 10131035).
13. 5-Fluorocytosine (Sigma-Aldrich, USA, Catalog # F7129).
14. TF1 cell line (ATCC, USA, Catalog # CRL-2003™).
15. Single-stranded DNA Oligonucleotides (Integrated DNA Technologies, USA).
16. Proteinase K Solution (Bioline, USA, Catalog # BIO-37084).
17. Buffer AL (Qiagen, Catalog # 19075).
18. DPBS, no calcium, no magnesium (Gibco, Catalog # 14190144).
19. QIAquick Spin Columns (Qiagen, Germany, Catalog # 28115).
20. 200 Proof Ethanol (Decon Labs, USA, Catalog # 2716).

21. Buffer AW1 (Qiagen, Germany, Catalog # 1067924).
22. Buffer AW2 (Qiagen, Germany, Catalog # 19072).
23. Nuclease-Free Water (Invitrogen, USA, Catalog # AM9937).
24. Phusion Flash High-Fidelity PCR Master Mix (Thermo Scientific, USA, Catalog # F548L).
25. Ethidium bromide solution (Sigma-Aldrich, USA, Catalog # E1510-10ML).
26. Gel Loading Dye, Purple (6X) (New England Biolabs, USA, Catalog # B7024S).
27. HyperLadder™ 100bp (Bioline, USA, Catalog # BIO-33056).
28. Buffer QG (Qiagen, Germany, Catalog # 19063).
29. 2-Propanol, Molecular Biology Grade, Fisher BioReagents™ (Fisher Scientific, USA, Catalog # BP2618500).
30. Buffer PE (Qiagen, Germany, Catalog # 19065).

Equipment

• 37°C, 5% CO2 incubator (Thermo Scientific, USA, Catalog # TH-3110).
• Cell culture microscope (Labomed, USA, Catalog # S96062).
• Cell counter (Bio-Rad, USA, Catalog # 1450102).
• Centrifuge (VWR, USA, Catalog # B30316).
• T100 Thermal Cycler (Bio-Rad, USA, Catalog # 1861096).
• Owl™ EasyCast™ B1A Mini Gel Electrophoresis Systems (Thermo Scientific, USA, Catalog # B1A-BP).
• UV Transilluminator (Accuris Instruments, USA, Catalog # 1177X75).
• NanoDrop™ One/OneC Microvolume UV-Vis Spectrophotometer (Thermo Scientific, USA, Catalog # ND-ONE-W).

Origin of plasmids for MEDS knock-in using CRISPR/Cas9 genome editing

1 For this protocol, two plasmids are required: a plasmid containing Cas9 endonuclease from the *Streptococcus pyogenes* Type II CRISPR/Cas system and single-guide RNA (sGRNA) and a plasmid that functions as the donor template for inserting the MEDS cassette into the β-globin (HBB) locus.

2 Acquire or create a plasmid that allows for the co-expression of SpCas9 and sgRNA to target the β-globin locus.

2.1 To target the human β-globin locus, the gRNA spacer sequence 5'-GTACACATATTGACCAAATC-3' was used. Alternatively, the gRNA spacer sequence 5'-TATGGGCAACCCTAAGGTGA-3' can be used.

3 Acquire the donor plasmid for knock-in of MEDS in the β-globin locus that contains the following functional elements: Left and right homology arms are used to direct the insertion into the β-globin locus.

3.1 Each homology arm consists of a 600 bp human β-globin locus-specific homologous sequence on either side of the MEDS functional cassette. The homologous sequence flanks the hemoglobin beta gene.

4 Note: The techniques outlined in this protocol can be used to knock-in additional transgenes for positive selection to the β-globin locus in human cells. In our case, neomycin phosphotransferase II was used, but other positive selection markers, including puromycin, blasticidin, hygromycin, or bleomycin resistance can be used to generate stable genome-edited human TF1 cells.

Origin of plasmids for HbS insertion using CRISPR/Cas9 genome editing

5 Acquire or create a plasmid that facilitates the co-expression of SpCas9 and sgRNA for targeting MEDS.

5.1 To target NPT II within the MEDS cassette, the gRNA spacer sequence 5' AAGCCAAGCACCGGATCGAG 3' was used. Alternatively, the gRNA spacer sequence 5' ATGATGGACACCTTCTCGGC 3' can be used.

6 Acquire the donor plasmid for knock-in of HbS. This plasmid contains the following functional elements: Left and right homology arms are used to direct the insertion into the β-globin locus. Each homology arm consists of a 600 bp human β-globin locus-specific homologous sequence on either side of the HbS sequence. The homologous sequence flanks the hemoglobin beta gene.

TF1 cell transfection

7 Count TF1 cells using an automated cell counter.

8 Seed $1x10^5$ cells per well in 1 mL Complete RPMI media in a 12-well plate. Note: the media used will be cell type dependent.

9 Incubate at 37°C.

10 When cells reach 60–70% confluency, transfect TF1 cells by adding a 50 µL mixture of pMEDS donor plasmid containing MEDS expression system flanked by homologous arms for recombination, pCas-Guide plasmid containing Cas9 and sg RNA, and FuGENE transfection reagent in Opti-MEM media. Each plasmid amount should be 1 µg. FuGENE transfection reagent:total plasmid DNA volume ratio should be around 3:1. Add 37°C prewarmed Opti-MEM media to make up the final 50 µL volume.

11 At 48 hours after transfection, collect the cells from each well in individual 15 mL tubes and centrifuge at 1k rpm for 5 minutes at room temperature.

12 Aspirate the old media and add 10 mL of fresh complete RPMI media to each tube.

13 Transfer all 10 mL of cells into a tissue culture flask.

14 Every 3 days, split the TF1 cells 1:5. That is, add 2 mL cells from the previous flask and 8 mL complete RPMI media to a new flask.

Antibiotic selection and limiting dilution for suspension cell lines

15 At passage 8 around ~80% confluency, collect all media containing suspension TF1 cells in a 15 mL tube.

16 Centrifuge at 1k rpm for 5 minutes.

17 Aspirate old media from 15 mL tube.

18 Add 10 mL selective media, which is complete RPMI with 1000 µg/mL G418 to the a new flask. For the second round of transfection and to eliminate cells that contain MEDS, add 2000 µg/mL 5-FC to a new flask. Note: G418 and 5-FC concentrations will vary depending on the cell line used. Therefore, it is essential to perform a drug kill curve prior to transfection to determine the appropriate dosage required to eliminate cells.

19 Continue to passage cells for 7 days, replacing media every 2-3 days.

20 After 7 days, collect all media from the flask containing suspension TF1 cells in a 15 mL tube.

21 Centrifuge at 1k rpm for 5 minutes.

22 Count TF1 cells using an automated cell counter.

23 Dilute the cells to a concentration of 0.5 cells per mL of selective media. This should result in approximately 1 cell per well in most wells of a 96-well plate.

24 Transfer the cells to a sterile reservoir.

25 Add 100 mL of the cell suspension per well in a 96-well plate using a multichannel pipette.

26 Incubate at 37°C.

27 Replace selective media every 2-3 days.

28 Once 70-80% confluency is reached in the wells, expand the cells to a 25 cm^2 cell culture flask and subsequently a 75 cm^2 tissue culture flask.

Preparation of genomic DNA

29 Note: Prepare a flask of both wild type and transfected TF1 cells.

30 Once 80% confluency is reached, collect entire flask of CRISPR/Cas9 edited TF1 cells in a 15 mL tube.

31 Centrifuge at 1k rpm for 5 minutes.

32 Aspirate old media.

33 Wash cell pellet with 5 mL 1x DPBS.

34 Centrifuge at 1k rpm for 5 minutes

35 Aspirate old media.

36 Repeat steps 33-35 two times for a total of three washes.

37 Resuspend cell pellet in 200 mL 1x DPBS. Add the cells and 20 mL of proteinase K to a new 1.5 mL tube.

38 Add 200 mL buffer AL to the 1.5 mL tube and vortex for 15 seconds.

39 Incubate the 1.5 mL tube for 10 minutes at 56°C.

40 Add 200 mL 100% ethanol to the 1.5 mL tube and vortex for 15 seconds to mix.

41 Add 800 mL to the spin column. Centrifuge at 10k rpm for 1 minute.

42 Add supernatant back to spin column. Repeat step 41 two times for a total of 3x binding.

43 Aspirate flow through and repeat steps 41 and 42 with the remaining mix.

44 Add 500 mL of wash buffer AW1 and centrifuge at 6k xg for 1 minute. Discard flow through.

45 Add 500 mL of wash buffer AW2 and centrifuge at 17.9k xg for 3 minutes. Discard flow through.

46 Centrifuge empty column at 17.9k xg for 2 minutes to remove excess buffer. Discard any remaining flow through.

47 Replace collection tube in the spin column with a 1.5 mL tube.

48 Add 100 mL of nuclease-free water to the column and incubate for 2 minutes at room temperature.

49 Centrifuge at 8k xg for 1 minute.

50 Add eluent back to column and repeat step 49 two times for a total of three times.

51 Nanodrop to measure genomic DNA concentration.

52 Store genomic DNA in -20°C.

Verification of genome editing

53 Genome editing is confirmed using PCR and sequencing.

54 Design PCR primers to verify genome editing. One primer should bind outside the homology arm and the other primer should bind within the MEDS cassette.

55 Prepare a PCR mix as indicated below:

56

A	B	C
Reagent	Final concentration	Volume for 1x (μL)
Phusion	1x	12.5 μL
Forward primer (100 μM)	1 μM	2.5 μL
Reverse primer (100 μM)	1 μM	2.5 μL
Genomic DNA	50-200 ng	
Nuclease-free water	N/A	

A	B	C
Total	N/A	25.0 µL

56.1 A total of 50-200 ng of genomic DNA can be used. In our case, we used 50 ng genomic DNA. Note: include a negative control from non-transfected (WT) TF1 cells.

57 Perform a PCR reaction in a thermocycler:

58

A	B	C	D
Step	Temperature	Time	Number of cycles
Initial denaturation	98°C	30 s	1
Denaturation	98°C	10 s	30
Annealing		30 s	
Extension	72°C	1 min	
Final extension	72°C	5 min	1
Hold		Forever	

58.1 The optimal annealing temperature and extension time may vary according to the primers used and PCR amplicon length, respectively.

59 Add 5 µL of 6x loading dye to each sample and load 25 µL on a 1% agarose gel (0.7g agarose in 70 mL TAE) containing 0.5 µg/mL ethidium bromide at 80 V for 1 hr. Include a suitable 100-base-pair ladder.

60 Verify PCR and excise bands of interest from the agarose gel with a clean, sharp razor blade and place in 1.5 mL tube.

61 After weighing out gel band, add 3x volumes of Buffer QG. For example, for a 100 mg excised band, add 300 L of buffer QG.

62 Incubate at 50°C for 10 minutes. During this incubation period, vortex the tube every 2-3 minutes to help dissolve the gel.

63 Add 1 volume of isopropanol to the sample and vortex to mix. For a 100 mg excised band, add 100 mL of isopropanol.

64 Add 750 mL of the mix to a spin column.

65 Centrifuge at 7.5k rpm for 1 minute. Add the mix back to the column.

66 Repeat steps 64 and 65, 2 times for a total of three binds.

67 Discard supernatant.

68 Repeat steps 64-67 with the remaining mix.

69 Add 500 mL Buffer QG to the spin column.

70 Centrifuge at 13k rpm for 1 minute.

71 Pour off supernatant from the collection tube.

72 Add 750 mL Buffer PE to the spin column and centrifuge at 13k rpm for 1 minute.

73 Pour off supernatant from the collection tube.

74 Centrifuge empty column at 13k rpm for 2 minutes to remove residual wash from the column.

75 Replace collection tube in the spin column with a 1.5 mL tube.

76 Add 30 mL nuclease-free water to the spin column.

77 Incubate at 37°C for 2 minutes.

78 Centrifuge at 13k rpm for 1 minute.

79 Add eluent back to column and repeat step 78, 2 two times for a total of three times.

80 Nanodrop to measure DNA concentration. Use nuclease-free water as a blank for the Nanodrop measurement.

81 Store DNA in -20°C or proceed to reamplification of the primary PCR product.

82 Reamplify the primary PCR product using a PCR mix as indicated below:

83

A	B	C
Reagent	Final concentration	Volume for 1x (µL)
Phusion	1x	12.5 µL
Forward primer (100 µM)	1 µM	2.5 µL
Reverse primer (100 µM)	1 µM	2.5 µL
Genomic DNA	50-200 ng	
Nuclease-free water	N/A	
Total	N/A	25.0 µL

Use 30 ng of gel-purified PCR product.

84 Run a PCR reaction using the following thermocycler conditions:

A	B	C	D
Step	Temperature	Time	Number of cycles
Initial denaturation	98°C	30 s	1
Denaturation	98°C	10 s	30
Annealing		30 s	
Extension	72°C	1 min	

A	B	C	D
Final extension	72°C	5 min	1
Hold		Forever	

84.1 The optimal annealing temperature and extension time may vary according to the primers used and PCR amplicon length, respectively

85 Repeat steps 61-81 to gel-extract and purify PCR product.

86 Confirm the presence of the intended genome edit using Sanger sequencing.
